# Development of outbred CD1 mouse colonies with distinct standardized gut microbiota profiles for use in complex microbiota targeted studies

**DOI:** 10.1038/s41598-018-28448-0

**Published:** 2018-07-04

**Authors:** Marcia L. Hart, Aaron C. Ericsson, K. C. Kent Lloyd, Kristin N. Grimsrud, Allison R. Rogala, Virginia L. Godfrey, Judith N. Nielsen, Craig L. Franklin

**Affiliations:** 1 Comparative Medicine Program, University of Missouri, Columbia, Missouri United States of America; 2 University of Missouri Metagenomics Center, University of Missouri, Columbia, Missouri United States of America; 3 Department of Veterinary Pathobiology, University of Missouri, Columbia, Missouri United States of America; 4 Mutant Mouse Resource and Research Center, University of Missouri, Columbia, Missouri United States of America; 5 Mouse Biology Program, University of California, Davis, California, United States of America; 6 Mutant Mouse Resource and Research Center, University of California, Davis, California, United States of America; 7 Department of Pathology and Laboratory Medicine, University of North Carolina, Chapel Hill, North Carolina United States of America; 8 National Gnotobiotic Rodent Resource Center, University of North Carolina, Chapel Hill, North Carolina United States of America; 9 Center for Gastrointestinal Biology and Disease, University of North Carolina, Chapel Hill, North Carolina United States of America; 10 Mutant Mouse Resource and Research Center, University of North Carolina, Chapel Hill, North Carolina United States of America; 11 Mouse Metabolic Phenotyping Center, University of California, Davis, California, United States of America

## Abstract

Studies indicate that the gut microbiota (GM) can significantly influence both local and systemic host physiologic processes. With rising concern for optimization of experimental reproducibility and translatability, it is essential to consider the GM in study design. However, GM profiles can vary between rodent producers making consistency between models challenging. To circumvent this, we developed outbred CD1 mouse colonies with stable, complex GM profiles that can be used as donors for a variety of GM transfer techniques including rederivation, co-housing, cross-foster, and fecal microbiota transfer (FMT). CD1 embryos were surgically transferred into CD1 or C57BL/6 surrogate dams that varied by GM composition and complexity to establish four separate mouse colonies harboring GM profiles representative of contemporary mouse producers. Using targeted 16S rRNA amplicon sequencing, subsequent female offspring were found to have similar GM profiles to surrogate dams. Furthermore, breeding colonies of CD1 mice with distinct GM profiles were maintained for nine generations, demonstrating GM stability within these colonies. To confirm GM stability, we shipped cohorts of these four colonies to collaborating institutions and found no significant variation in GM composition. These mice are an invaluable experimental resource that can be used to investigate GM effects on mouse model phenotype.

## Introduction

The gastrointestinal tract is densely populated with myriad microorganisms including eukaryotes, prokaryotes, viruses, and fungi^1^. Largely unculturable, the gut microbiota (GM) has only recently been characterized as cost-effective high-throughput sequencing technologies such as whole genome and targeted sequencing have become available. With the development of these new technologies, there has been an explosion of studies demonstrating that alterations in gut microbial communities have both local and systemic physiologic effects in a variety of health and disease conditions^2–5^.

To study the GM, several methods of experimental manipulation in rodents can be performed dependent on experimental design needs^6–8^. These methods can be grouped into two main categories: 1) simple, defined GM methods - including the use of axenic, mono-associated, defined simple microbiota (e.g. altered Schaedler flora), or xenografted mice and 2) complex, undefined GM methods – including cross-fostering, co-housing, fecal microbiota transfer, or surgical embryo transfer. The use of axenic, mono-associated, and defined simple GM using a reductionist approach offers the opportunity to study host-microbe interactions by purposefully introducing defined microbial populations. These models rely on the reconstitution of axenic mice known to have abnormalities in several physiologic systems including an underdeveloped immune system^9–11^, epithelial turnover and metabolism^12–14^, and behavior^15^ which may complicate interpretation of study findings. An alternative strategy, is the use of xenobiotic mouse models whereby axenic mice are colonized with GM isolated from other species. While highly useful, studies indicate not all xenobiotic GM species can colonize mice, suggesting that these models may also have limitations^16,17^. It should also be noted, that colonization of axenic mice by any of the methods described above generally occurs at either weaning or adulthood thereby missing early life events dependent on early exposure to maternal GM *in utero* or at birth. Such mice are thus best employed after a generation of breeding.

Complex GM transfer methods offer an attractive addition to more simplified GM studies by providing an opportunity to investigate naturally occurring host-microbe and microbial community interactions more reflective of those occurring naturally in both health and disease. Microbiota transfer methods such as cross-fostering and co-housing are cost-effective and can easily be performed in the vast majority of laboratories. However, cross-fostering requires careful consideration for timing of pup transfer to surrogate dam and housing environment as cannibalism of newly transferred pups can occur. Co-housing mice can be an alternative strategy wherein, mice of the desired model are housed with donor mice colonized by a differing GM profile. Incomplete GM transfer can occur, resulting in a hybrid GM with contributions from both recipient and donor^18^ which may or may not be desirable, dependent on mouse model and study design. Methods such as fecal microbiota transfer (FMT) can be utilized to colonize weanlings or adult mice, previously given an antibiotic cocktail, with complex fecal material isolated from a GM donor. Relying heavily on antibiotic effectiveness, this method can also result in incomplete transfer and may be confounded by differences in GM complexity of both recipient and donor^19^. Cross-fostering, co-housing and FMT offer great options for complex GM transfer. However, as with more simplified reductionist GM approaches, these methods do not provide GM exposure *in utero* or immediately at birth thereby requiring additional breeding generations for the study of early life events.

Surgical embryo transfer offers a desirable alternative strategy to study the effects of complex GM at all stages of development. Embryos or morulae of the desired mouse model are surgically implanted into conventionally housed surrogate dams with the desired GM profile. We have previously demonstrated that GM composition and complexity can vary by animal producer^20^ and can alter disease phenotype^21,22^. Unfortunately, the desired GM profile may not be available in the chosen mouse model and is often associated with inbred mouse strains that have low maternal care instincts and thusly, low reproduction indices. In addition, inbred mice often have decreased success rates for embryo transfer and pup survival, making them suboptimal as surrogate dams. In order to overcome this, we hypothesize that outbred colonies of CD1 mice could be generated with pre-defined mouse producer specific GM profiles. We demonstrate that CD1 colonies can indeed be generated with specific GM profiles that are stable and can be maintained for at least nine generations. Due to the strong maternal care instincts of these mice and the stability of the established GM profiles, these mice are invaluable tools for targeted complex microbiota studies and provide an additional strategy for GM colonization.

## Materials and Methods

### Mice

The current study was conducted in accordance with the guidelines set forth by the Guide for the Use and Care of Laboratory Animals, the Public Health Service Policy on Humane Care and Use of Laboratory Animals, and were approved by the University of Missouri Institutional Animal Care and Use Committee, University of California at Davis Institutional Animal Care and Use Committee, and the University of North Carolina at Chapel Hill Institutional Animal Care and Use Committee.

For embryo transfer (ET) recipients, eight to ten week-old female C57BL/6J (The Jackson Laboratory, Bar Harbor, ME facilities), C57BL/6NTac (Taconic Biosciences, Inc., Cambridge City, IN facilities), Crl:CD1 (Charles River Laboratories, Kingston, NY facilities), and Hsd:ICR (Envigo Corporation, Indianapolis, IN facilities) mice were purchased and allowed to acclimate for one week prior to use. Vasectomized, seven to eight week-old Crl:CD1 male mice (Charles River Laboratories) were co-housed with surrogate embryo recipient females (C57BL/6J, The Jackson Laboratory; C57BL/6NTac, Taconic Biosciences, Inc., Cambridge City, IN facilities; Crl:CD1, Charles River Laboratories; and Hsd:ICR, Envigo Corporation, Indianapolis, IN facilities) to induce pseudopregnancy and intrauterine embryo transfer was performed as described below. All mice were housed in microisolator cages on ventilated racks (Thoren, Hazelton, PA) with autoclaved pelleted paper chip bedding (Shepherd Specialty Papers, Watertown, TN), placed on a 14:10 light-dark cycle, and provided *ad libitum* access to 5058 irradiated breeder chow (LabDiet, St. Louis, MO) and acidified autoclaved water.

### Embryo collection and transfer

On day 1, 5 week-old female Crl:CD1 embryo donors (Charles River Laboratories, Kingston, NY facilities) received an intraperitoneal (IP) injection of 5 IU of pregnant mare serum gonadotropin (PMSG) (Calbiochem, San Diego, CA) in 0.2 ml Dulbecco’s phosphate-buffered saline (DPBS) with no calcium or magnesium (Life Technologies, Carlsbad, CA) at 2.5 hours post-light induction to induce superovulation. On day 3, at 5 hours post-light induction, embryo donors received an IP injection of 5 IU human gonadotropin (hCG) in 0.2 ml DPBS and were mated to intact males of the same genotype. Post-mating, CD1:CRL embryo donors were euthanized and embryos at the zygote cell stage were collected aseptically. Briefly, the skin and surrounding fur was soaked in 70% ethanol and the peritoneal cavity was opened for visualization of the reproductive tract. Using aseptic surgical techniques, the oviducts were excised and placed in 50 µl of sterile pre-warmed type IV-S hyaluronidase (Sigma, St. Louis) reconstituted at 1 mg/ml in HEPES media (Sigma) supplemented with 4 mg/ml bovine serum (Sigma) for five to ten minutes. Oviducts were visualized using a dissecting microscope and clutches of zygotes were released from oviducts with gentle manipulation and collected with a sterile glass hand-pipette. To coincide with embryo collection from donor females, surrogate embryo recipient females (C57BL/6 J, The Jackson Laboratory; C57BL/6NTac, Taconic Biosciences, Inc., Cambridge City, IN facilities; Crl:CD1, Charles River Laboratories; and Hsd:ICR, Envigo Corporation, Indianapolis, IN facilities) demonstrating uninduced signs of estrus were mated on day 3 with a sterile, vasectomized Crl:CD1 male (Charles River Laboratories). Surrogate females were inspected for copulatory plugs and plug-positive mice were used for embryo transfer as previously described^22^. Briefly, surrogate females were anesthetized via IM injection of ketamine/xylazine cocktail at 5.5 mg and 1 mg per 100 g body weight, placed in sternal recumbency, the dorsum shaved, and loose hair removed by wiping the shaved area with gauze moistened in 70% ethanol. To prepare for surgery, the surgical area was scrubbed three times using an alternating pattern of betadine soaked cotton swabs followed by 70% ethanol soaked cotton swabs. Once the surgical site was prepared, a dorsal midline incision was made and the uterine oviducts located by dissecting through the retroperitoneal muscle. Zygotes in 3 to 5 µl of media were injected into the oviducts using a glass hand-pipette. Skin incisions were closed with sterile surgical staples and mice received a subcutaneous injection of 2.5 mg/kg of body weight flunixin meglumine (Banamine®) prior to recovery on a warming pad.

### Establishment and maintenance of GM colonies

Offspring from embryo transfers were housed with their recipient dam until weaning (21 days of age). To reduce possible confounds of differences in litters, weanling mice were randomly separated into cages of 4 mice, dependent on GM profile and sex, and raised to adults. Adult male and female mice (8–10 weeks of age) with the same GM profile were chosen for mating using a random number generator to establish a randomized outbred mating pattern, ensuring no related individuals were mated, and colonies were maintained for ongoing generations. All mice were handled using standard barrier housing techniques Briefly, all personnel working with the GM colonies were required to wear hair bonnet, disposable gown, face mask, and double gloves before entering the room. Mice were handled in order of ascending GM complexity (e.g., GMJAX, GMTAC, GMCRL, GMHSD) with only one GM profile handled at a time. All cages were opened under a class BSL2 ventilated hood and outer gloves were sprayed with a freshly made 10% bleach solution before touching contents inside the cage (e.g., mice, food, water, or bedding). Outer gloves were resprayed with bleach before handling each subsequent cage. For routine cage changes, mice were grasped at the base of the tail using bleached forceps and placed in a new autoclaved cage. Outer gloves were changed before handling each new GM profile.

All mice were housed in autoclaved microisolator cages on individually ventilated racks (Thoren, Hazelton, PA) with autoclaved pelleted paper chip bedding (Shepherd Specialty Papers, Watertown, TN), autoclaved cotton fiber nestlet (Ancare, Bellmore, NY), placed on a 14:10 light-dark cycle, provided *ad libitum* access to 5058 irradiated breeder chow (LabDiet, St. Louis, MO) and acidified autoclaved water. Each GM colony was housed in order of ascending GM complexity (described above) with each GM colony occupying one side of two designated IVC racks. Other research colonies with the same health status were maintained in the room on separate IVC racks. These mice received cage changes either after the CD1 colonies were changed or were changed on different days. All research mice in the room were handled adhering to the same bleach protocol as described above.

### Mice shipped to collaborating institutions

Six week-old female mice from the four GM colonies were grouped and placed in autoclaved, plastic 22” × 16” × 7” Taconic Transport cages mouse shipping container (Taconic Biosciences, Inc., Cambridge City, IN) containing autoclaved pelleted paper chip bedding (Shepherd Specialty Papers, Watertown, TN) and gel packs (Napa-Nectar, Systems Engineering, Napa, CA). Shipping procedures were in accordance with IACUC guidelines and the Mutant Mouse Resource and Research Center (MMRRC) mouse shipping policies. Mice were received by the shipping carrier the morning of packaging and arrived at collaborating institutions within a 24 hour period. Following arrival, mice were unpacked, housed as 4 per cage, and grouped according to GM profile. Barrier housing conditions, feed, and bedding were chosen by collaborating institutions. All cages were handled in order of ascending GM complexity (e.g. GMJAX, GMTAC, GMCRL, GMHSD) to minimize possible GM cross contamination. Fecal samples were collected at 24 hours pre-shipping and following arrival (day 0) and acclimation (day 14) at collaborating institutions.

Mice housed at the University of North Carolina (UNC) were housed at a density of four mice per cage in autoclaved cages on racks with individually ventilated cages (Techniplast Greenline IVC, West Chester, PA) lined with irradiated Teklad corncob bedding (Envigo Corporation, Indianapolis, IN). Mice were given *ad libitum* access to autoclavable Teklad 2020 rodent diet (Envigo Corporation, Indianapolis, IN), autoclaved reverse osmosis purified water, and kept on a 12:12 day-night light cycle. Mice housed at the University of California at Davis (UCD) were housed at a density of four mice per cage in autoclaved, individually ventilated cages on Optimice racks (Animal Care Systems, Inc., Centennial, CO) lined with 1/8” autoclaved corn cob bedding. Mice were given *ad libitum* access to irradiated Teklad 2918 rodent diet (Envigo Corporation, Indianapolis, IN), autoclaved acidified water, and kept on a 12:12 day-night light cycle.

### Fecal collection and isopropanol DNA extraction

Six to eight week-old mice were placed in an empty autoclaved cage and two freshly evacuated fecal pellets were placed in a sterile 2 ml Eppendorf round-bottom tube (Thermo Fisher Scientific, Pittsburgh, PA) containing a sterile 0.5 cm diameter stainless steel bead as previously described^20^. Samples were stored at −80 °C until processed for DNA extraction. For DNA extraction, lysis buffer was added (800 µl per tube) and samples were mechanically disrupted using a TissueLyser II (Qiagen, Venlo, Netherlands) for 3 minutes at 30 Hz, followed by incubation at 70 °C for 20 minutes with periodic vortexing. Samples were centrifuged at 5000 × g for 5 minutes, and the supernatant was transferred to a sterile 1.5 ml Eppendorf tube containing 200 µl of 10 mM ammonium acetate. Lysates were vortexed, incubated on ice for 5 minutes, and then centrifuged. Supernatant was transferred to a sterile 1.5 ml Eppendorf tube and one volume of chilled isopropanol was added. Samples were incubated on ice for 30 minutes and centrifuged at 16000 × g at 4 °C for 15 minutes. The DNA pellet was washed with 70% ethanol and resuspended in 150 µl Tris-EDTA, followed by addition of 15 µl of proteinase K and 200 µl AL buffer (DNeasy Blood and Tissue kit, Qiagen). Samples were incubated at 70 °C for 10 minutes and 200 µl of 100% ethanol was added to the tubes. Samples were mixed by gentle pipetting and the contents transferred to a spin column from the DNeasy kit (Qiagen). The DNA was further purified following the manufacturer’s instructions and eluted in 200 µl EB buffer (Qiagen). DNA concentrations were determined fluorometrically (Qubit dsDNA BR assay, Life Technologies, Carlsbad CA) and samples were stored at 20 °C until sequencing.

### Library Construction and 16S rRNA sequencing

Library construction and sequencing was performed at the University of Missouri Metagenomics Core. Bacterial 16S rRNA amplicons were generated in a multiplexed (96-well) format using amplification of the V4 hypervariable region of the 16S rRNA gene, and then sequenced on the Illumina MiSeq platform as previously described^20,23^. All samples for this study had an average of greater than 96,000 reads and were deemed to have successful amplification.

### Informatics analysis

Read merging, clustering, and annotation of DNA sequences was performed by the University of Missouri Informatics Research Core Facility. Briefly, paired DNA sequences were merged using FLASH software^24^, and removed if found to be far from the expected length of 292 after trimming for a base quality less than 31. Cutadapt^25^ (https://github.com/marcelm/cutadapt) was used to remove the primers at both ends of the contig and reject contigs that did not contain both primers. The usearch^26^ fastq_filter command (http://drive5.com/usearch/manual/cmd_fastq_filter.html) was used for quality trimming of contigs, rejecting those for which the expected number of errors was greater than 0.5. All contigs were clipped to 248 bases and shorter contigs rejected. The Qiime v1.9^27^ command split_libraries_fastq.py was used to demultiplex the sample. The outputs for all samples were concatenated into one file for clustering. The uparse^28^ method (http://www.drive5.com/uparse/) was used to both cluster contigs with 97% similarity and remove chimeras. Taxonomy was assigned to selected OTUs using BLAST^29^ against the SILVA database v128^30^ of 16SrRNA sequences and taxonomy.

### Statistical Analysis

For gut microbiota analysis, bar graphs were generated with Microsoft Excel (Microsoft, Redmond WA) and principal coordinate analysis (PCoA) was generated using Paleontological Statistics Software Package (PAST) 3.12^31^. All groups were visually inspected for descriptive analysis of consistency between animals (bar graphs) or clustering of animals within groups by principal coordinate analysis (PCoA). Statistical testing for differences in beta-diversity was performed via two-way PERMANOVA, implemented using PAST 3.12. Specifically, main effects of and interactions between GM source and generation or institution, respectively, were determined using two-way PERMANOVA, and pairwise comparisons between groups were performed using one-way PERMANOVA of the same groups. Statistical analysis of differences in family relative abundance was performed by one-way ANOVA or Kruskal–Wallis, depending on normality of data as determined via Shapiro-Wilk normality testing, using GraphPad Prism 7.0 for Windows (GraphPad Software, La Jolla, CA, www.graphpad.com) with GM profile (i.e., GMJAX, GMTAC, GMCRL, GMHSD) as the independent variable. Statistical significance of families were assumed if *p* ≤ 0.05, taking into account false discovery rate using the original method of Benjamini-Hochberg correction for multiple testing^32^. Hierarchical clustering and statistical significance at the operational taxonomic unit (OTU) level was determined by one-way ANOVA using MetaboAnalyst 3.0^33,34^.

## Results

### Generational CD1 offspring have GM profiles similar to embryo transfer surrogate dams

**Figure 1:**
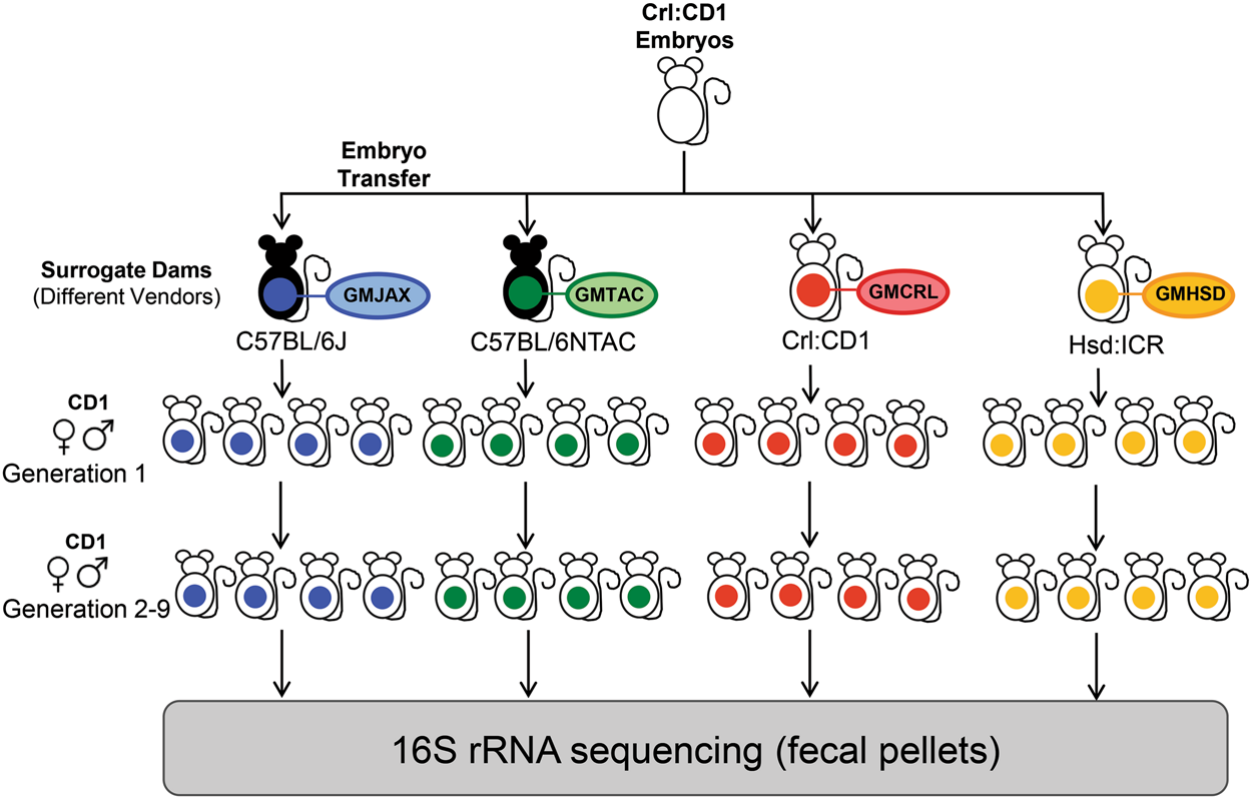
Experimental design used to generate CD1 mice with four different gut microbiota (GM) profiles. Schematic diagram showing embryo transfer scheme used to rederive CD1 mice to C57BL/6J_GMJAX_, C57BL/6NTac_GMTAC_, Crl:CD1_GMCRL_, and HSD:ICR_GMHSD_ surrogate dams. At maturity, offspring were mated using an outbred mating scheme within each GM profile and maintained as four separate breeding colonies for nine generations.

It is well-established that in mammals the gastrointestinal tract of offspring is colonized by maternal microbiota during and shortly following the birthing process. We chose to capitalize on this phenomenon to generate outbred CD1 colonies harboring distinct GM profiles that are naturally occurring in contemporary rodent producers. To this end, adult female C57BL/6J (The Jackson Laboratory), C57BL/6NTac (Taconic Biosciences, Inc), Crl:CD1 (Charles River Laboratories), and Hsd:ICR (Envigo) mice colonized with GM profiles varying in complexity and composition were used as recipients for surgical embryo transfer (ET) of CD1 embryos (Fig. 1). Following birth, pups remained with the surrogate dam until weaning, and were raised to adulthood in ventilated micro-isolator racks following strict barrier husbandry practices to minimize GM cross contamination. Fecal samples from 6–8 week-old first, second, and ninth-generation offspring were characterized via sequencing of the V4 hypervariable region of the 16S rRNA gene.

**Figure 2:**
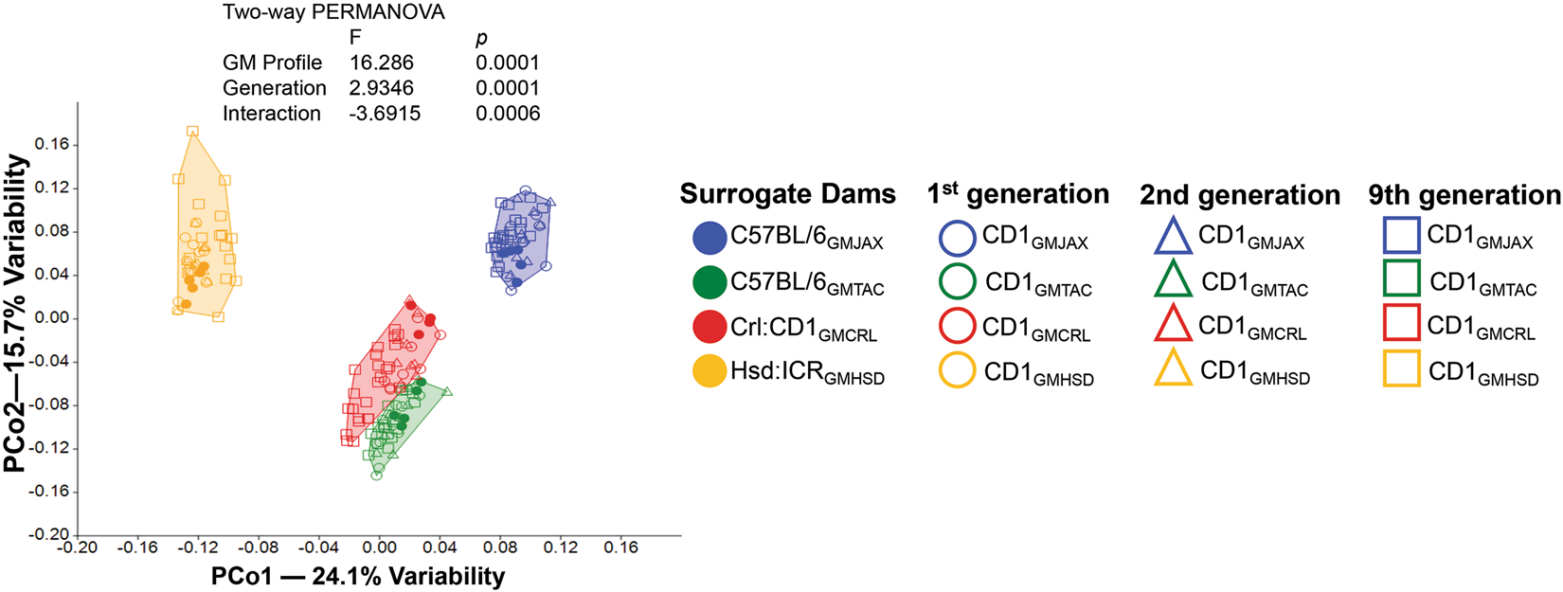
First, second, and ninth generation females maintain four distinct gut microbiota (GM) communities similar to surrogate dams. Principal coordinate analysis (PCoA) of representative fecal samples from surrogate dams (n = 5) and 6 to 8 week-old CD1 female offspring, first and second generation (n = 8–10) and ninth generation (n = 18–20). Figure legend of GM and generational groups located right of PCoA. Statistical significance determined using two-way PERMANOVA (*p* ≤ 0.05 statistically significant).

To evaluate GM composition of generational offspring as compared to ET surrogate dams, we performed principal coordinate analysis (PCoA) at the OTU level. In PCoA, samples that are similar in microbial composition cluster together, whereas samples that are dissimilar are farther apart. As anticipated, generational offspring were colonized with similar GM profiles to surrogate dams and had intragroup clustering indicating similar β-diversity (Fig. 2). In addition, differential intergroup clustering into four distinct groups illustrates the presence and maintenance of four distinct GM profiles regardless of surrogate dam and generation. Interestingly, mice harboring Taconic GM (hereafter referred to as GMTAC) and Charles River GM (GMCRL) showed similar intergroup clustering along PCo1 indicating a greater similarity between these two GM profiles than either GMJAX (The Jackson Laboratory) and GMHSD (Envigo). Despite similar clustering along PCo1, there is however separation between GMTAC and GMRCL along PCo2 demonstrating two distinct GM profiles (*p* ≤ 0.001 as determined by Two-way PERMANOVA). Broken-stick analysis of coordinates revealed a greater-than-expected contribution to total variance extending to PCo8 (data not shown), although there was a clear break between PCo2 and PCo3 suggesting the bulk of the observed variation is captured along coordinates PCo1 and PCo2. Visual inspection of 2d plots comparing PCo1 or PCo2 to all subsequent components through PCo8, failed to reveal any further clustering. Taken together, these data indicate generational stability of the GM, with the majority of observed variation attributed to source of surrogate dam and the associated producer-specific GM (24.75%) and lesser contribution to variation by generation (4.46%), as determined by the sum of squares associated with each fixed variable in the two-way PERMANOVA. These data suggest that the tested GM profiles are stable and can be maintained under the current animal housing and husbandry conditions.

### Ninth generation CD1 colonies maintain four distinct GM profiles varying in composition and complexity

**Figure 3:**
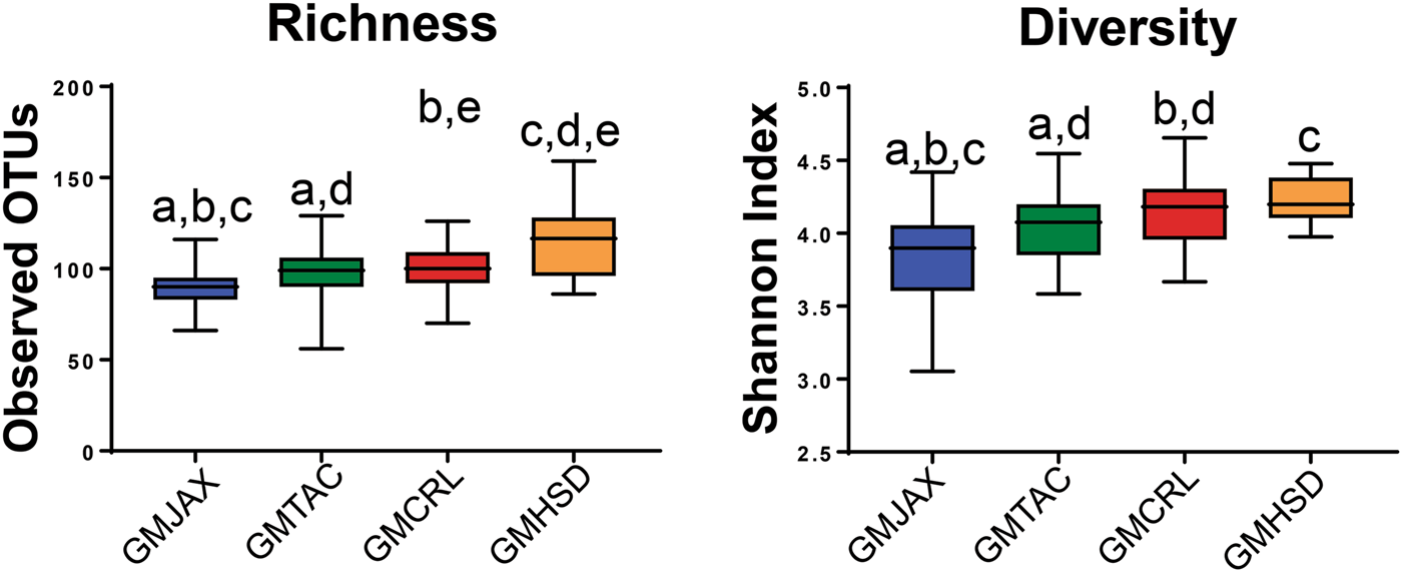
Ninth generation offspring maintain distinct GM profiles varying in richness and diversity. Number of observed OTUs and Shannon estimate of microbial diversity plotted by Tukey box and whisker graph of ninth generation female mice (n = 18–20 per GM profile). Statistical significance determined using one-way ANOVA (*p* ≤ 0.05 statistically significant). Statistical significance between groups annotated by same lower case letters above box plots.

**Figure 4:**
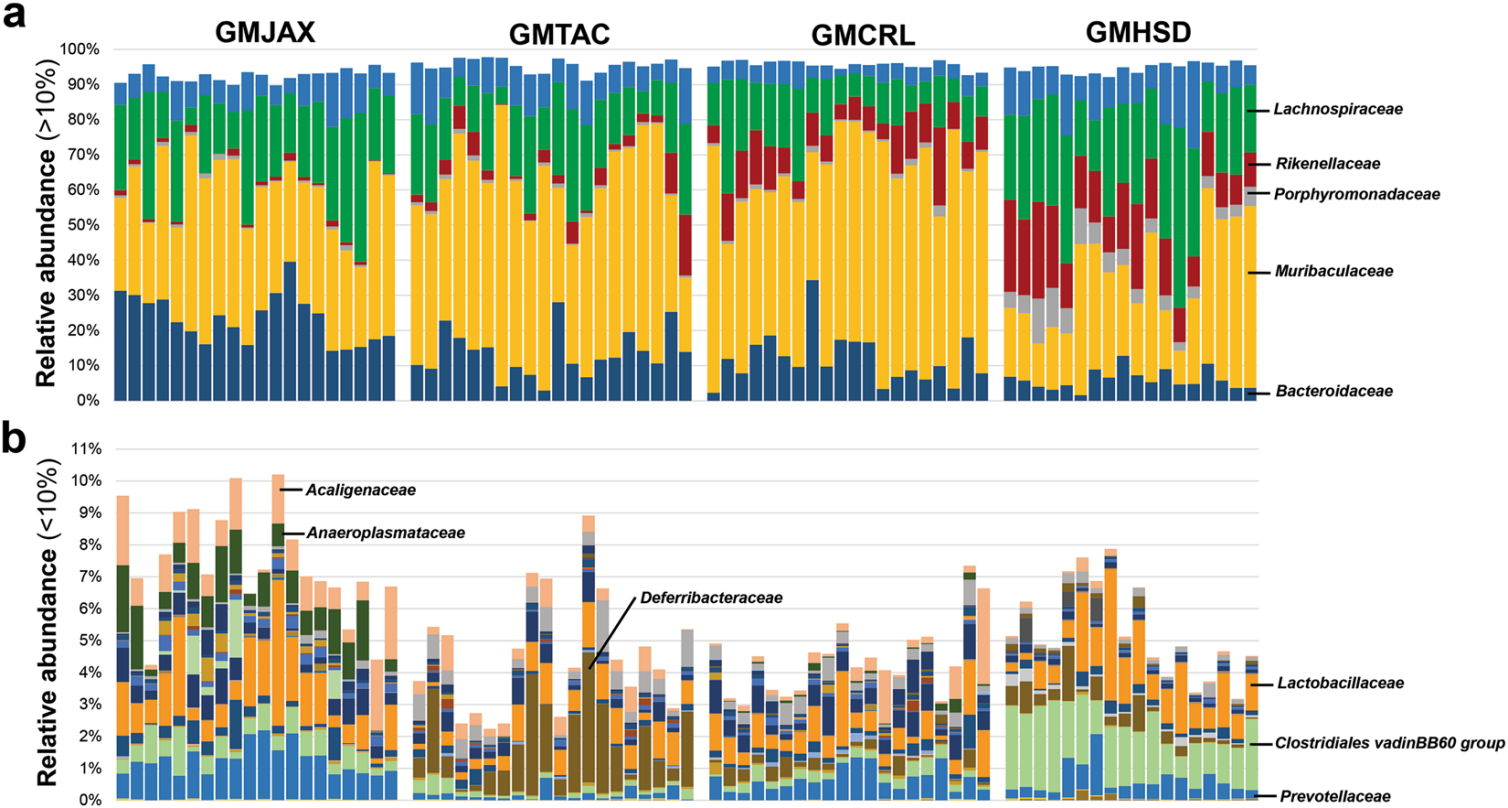
Ninth generation CD1 mice differ in GM composition in accordance with producer origin of embryo transfer surrogates. Representative samples for ninth generation 6 to 8 week-old CD1 female mice (n = 18–20 per GM profile). (**a**) Bar charts of relative abundance of taxa at family level with a group mean relative abundance representing greater than 10%. (**b**) Bar charts of relative abundance of taxa at family level with a group mean relative abundance representing less than 10%. Statistical significance determined using one-way ANOVA or Kruskal–Wallis, depending on normality of data as determined via Shapiro-Wilk normality testing, and Benjamini-Hochberg correction for multiple testing (*p* ≤ 0.05 statistically significant). Statistically significant families noted by taxonomic name on bar chart.

**Figure 5:**
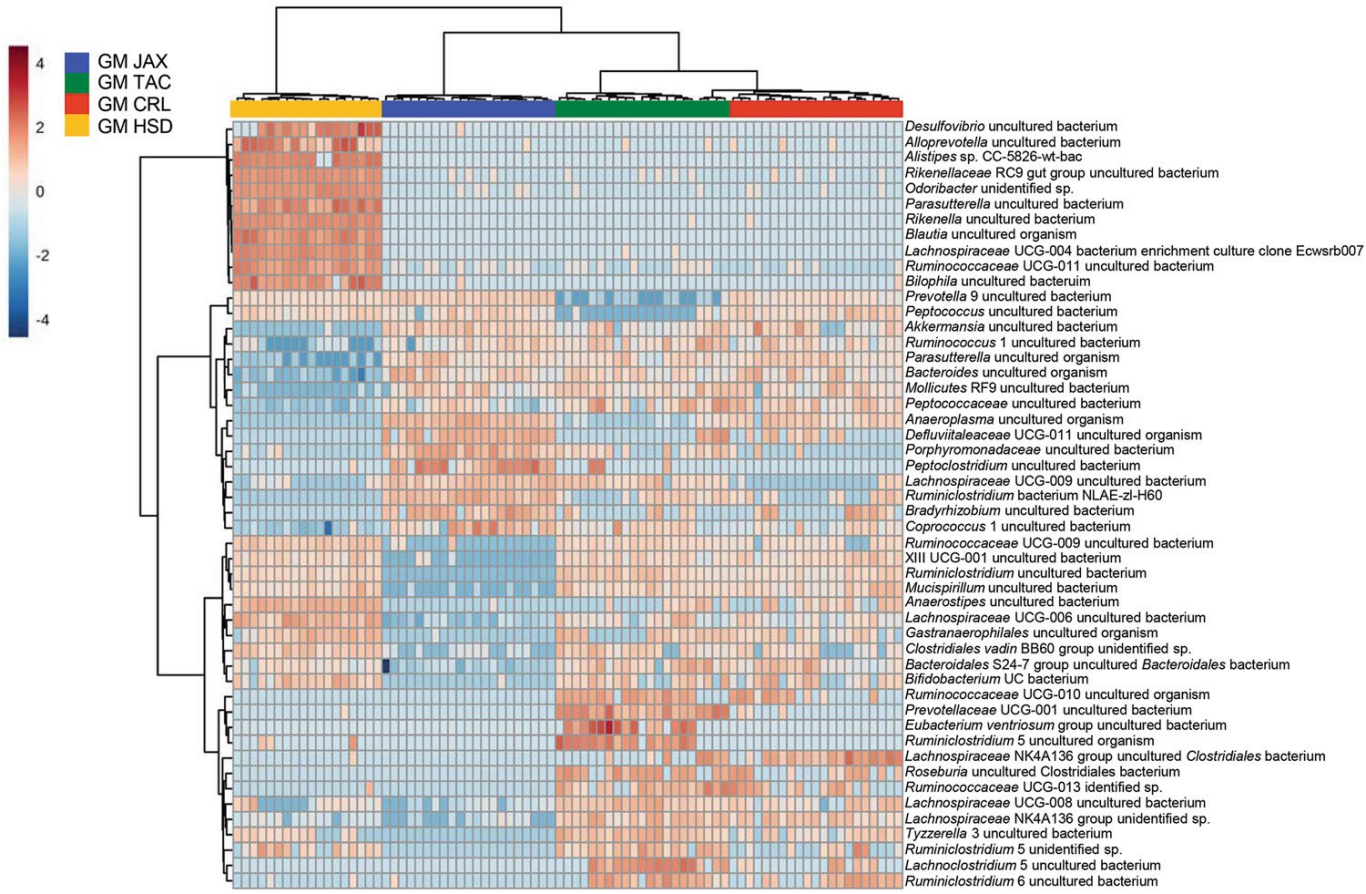
Hierarchical cluster analysis of ninth generation fecal samples. Hierarchical cluster analysis of the top 50 statistically significant operational taxonomic units (OTUs) in each GM profile. Color intensity indicates log_2_ normalized abundance of OTUs in each sample. Color coded bars at top indicate GM profile.

To evaluate the potential use of these colonies as complex GM donors for future microbiota targeted experiments, we examined the composition and complexity of 6–8 week old ninth generation female offspring. Comparison of bacterial richness between GM profiles revealed lower richness in GMJAX females compared to all other GM profiles (Fig. 3). Conversely, GMHSD mice had the greatest richness compared to other groups. Bacterial α-diversity between groups was evaluated using the Shannon index. Similar to our richness findings, GMJAX females had the lowest α-diversity as compared with the other groups (Fig. 3). To characterize the taxonomic differences in GM composition between each colony, statistical tests were performed using the relative abundance data annotated to the level of family. Notably, we found several families that differed between each GM profile (Fig. 4a,b). Specifically, we found that CD1 mice with the GMHSD profile has significantly higher relative abundance of families *Rikenellaceae* (mean relative abundance ±SD = 15.9%, ±0.06), *Porphyromonadaceae* (11.2%, ±0.03), and *Clostridiales vadin*BB60 group (1.76%, ±0.006) as compared to the other GM profiles. Conversely, we found that mice with the GMJAX profile had increased relative abundance of *Bacteroidaceae* (29.29%, ±0.06), *Alcaligenaceae* (1.04%, ±0.006), *Anaeroplasmataceae* (1.01%, ±0.004), and *Prevotellaceae* (1.24%, ±0.004). Mice harboring the GMTAC profile had an increased abundance of *Deferribacteraceae* (1.5%, ±0.001) as compared to other groups. Both GMJAX and GMHSD had similar levels of *Lachnospiraceae* (22.8%, ±0.09 and 26.6%, ±0.08, respectively) and *Lactobacillaceae* (1.3%, ±0.006; and 1.2%, ±0.006, respectively), that were greater than those with the GMTAC or GMCRL profiles. Additionally, GMTAC and GMCRL had increased abundance of *Muribaculaceae* (49.4%, ±0.139; and 54.6%, ±0.118) as compared to mice colonized with the GMJAX or GMHSD profiles. When annotated to the level of OTU, hierarchical cluster analysis demonstrated clustering of GMCRL and GMTAC profiles suggesting some similarity at the OTU level (Fig. 5). Interestingly, there was extensive patterning between GMHSD and GMJAX profiles with increased relative abundance of similarly clustered OTUs in mice colonized with GMHSD that were below detectable levels or with decreased relative abundance in the GMJAX group suggesting that these two profiles are dramatically different in composition.

### Ninth generation CD1 colonies maintain stable GM profile following shipping

**Figure 6:**
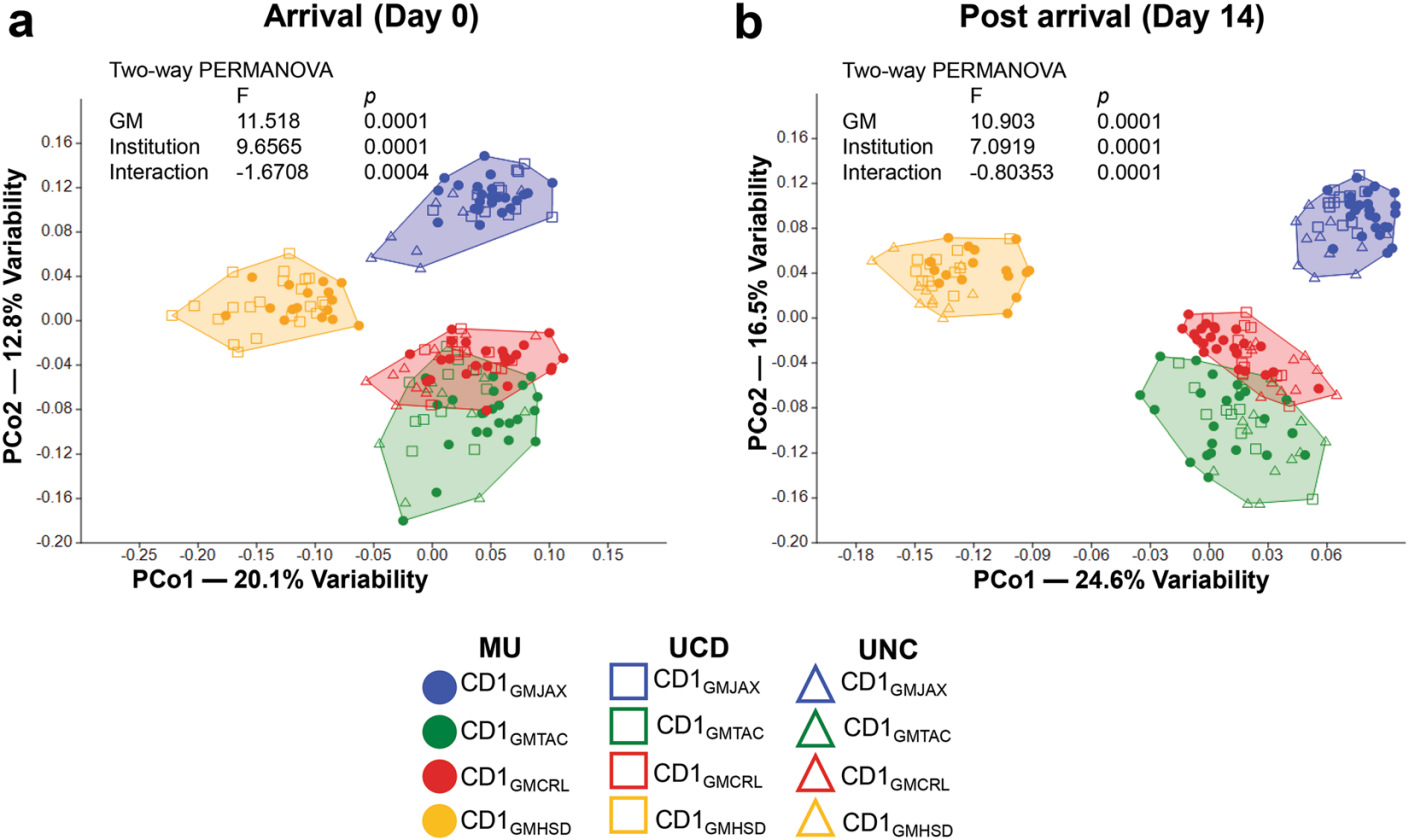
Ninth generation CD1 females maintain consistent GM profile after shipping to new institution. Principal coordinate analysis (PCoA) of representative fecal samples from 6 week-old, ninth generation CD1 females (**a**) at arrival at new institution and (**b**) 14 days post shipping (n = 24 pre-shipment samples collected at MU; n = 12 post shipment samples collected at UCD and UNC on day 0 and day 14 post shipment). Figure legend of GM profile and institution located below PCoA; MU = University of Missouri, UCD = University of California Davis, and UNC = University of North Carolina. Statistical significance determined using two-way PERMANOVA (*p* ≤ 0.05 statistically significant).

Mice with fully characterized, stable GM profiles offer a unique resource to study complex GM communities. However, the utility of these mice is limited if the GM profiles cannot be used by other research groups. In order to investigate the stability of these GM profiles at other institutions, we shipped six week-old female mice from each of the four colonies to two collaborating institutions. Pre-shipment fecal samples were taken from all mice at 24 hours before packing and shipping. Additional fecal samples were taken at collaborating institutions at arrival (day 0) and 2 weeks post arrival (day 14). To prevent possible GM cross contamination, mice were housed under barrier conditions and handled in order of ascending GM complexity as previously described (i.e., GMJAX, GMTAC, GMCRL, GMHSD). All other housing conditions, including bedding and diet, were at the discretion of collaborating institutions. Based on visual inspection of PCA and testing via PERMANOVA, we found that the four GM profiles were maintained at each institution regardless of fecal time point collection and differences in housing conditions (Fig. 6a,b) demonstrating the stability of GM profile. Interestingly, within each GM profile there were shifts in intra-GM clustering of samples from each institution along PCo1 (day 0) and along PCo1 and PCo2 (day 14) dependent on day of collection, with GMCRL and GMTAC profiles demonstrating the most dramatic shifts in clustering along PCo2 (day 0 and day 14). This suggests that some differences in GM composition likely occur immediately post shipping with GM stabilization occurring after acclimation to the new institution. Regardless, the observed shifts in clustering did not affect overall maintenance of four distinct profiles. Broken-stick analysis of coordinates revealed a greater-than-expected contribution to total variance extending to PCo7 for both pre- and post- arrival time points (data not shown). However, in alignment with our previous observations, there was a clear break between PCo2 and PCo3 suggesting the bulk of the observed variation is attributed to coordinates PCo1 and PCo2. Visual inspection of 2d plots comparing PCo1 or PCo2 to all subsequent components through PCo7, failed to reveal any further clustering. This suggests that the majority of observed variation can be attributed to producer-specific GM (16.47% at day 0; 15.64% at day 14) with lesser institutional contribution (9.20% at day 0; 6.78% at day 14) indicating stability of GM profile post shipping.

## Discussion

As interest in the study of the gut microbiota continues to rise, there is an increasing need for methods to experimentally manipulate microbial composition and function. Current systems such as germ-free mice or mono-colonized mice do not account for interactions between microbes in naturally occurring complex gastrointestinal populations and, with the exception of embryo transfer studies, do not account for the impact of exposure to the GM during early development. Human studies, while useful for studying complex microbe interactions are often confounded by uncontrollable experimental variables and have limited longitudinal utility due to collection of GM samples after disease onset. Therefore, animal models such as mice with translatable application are essential to propel the gut microbiome field forward.

One solution to encompass study of GM effects at early time points is to use transfer of embryos with the desired genotype to surrogate dams with differing GM profiles. However, the GM profiles of interest are often associated with inbred mouse strains. Such mice often have decreased reproductive indices relative to outbred stocks, making their use as surrogate dams difficult. To overcome these limitations, we investigated the use of embryo transfer as a method to develop outbred mouse colonies colonized with distinct producer-specific gut microbiota (GM) profiles. We surgically implanted embryos from the same outbred stock into surrogate dams harboring different GM communities varying in complexity and composition. We found that first, second, and ninth generation offspring were similar in β diversity to surrogate dams indicating successful transfer of complex GM profile from dam to pup.

Complex microbiota targeted studies can be a valuable translatable resource for study of GM effects on health and disease. Anecdotally, it has been suggested that stable, complex GM profiles cannot be successfully maintained. In order to use these colonies as surrogate dams for embryo transfer, they must exhibit longevity and stability of GM composition in a breeding colony. We found that by using standard barrier husbandry techniques, breeding colonies with distinct GM profiles are maintained with minimal GM drift for at least nine generations. Furthermore, we found that shipped mice from these colonies maintained similar GM profiles at collaborating institutions. We acknowledge that there is potential for different results if the husbandry at a receiving institution were sufficiently disparate from that at the receiving institution. However, this study underscores that with appropriate husbandry procedures, stable GM profiles can be maintained in healthy mice at any institution.

Embryo transfer provides an alternative strategy to evaluate the contribution of complex GM in both health and disease and encompasses early life colonization. The outbred CD1 colonies we generated have stable GM profiles with demonstrable differences in both composition and complexity. These mice provide an opportunity to study the effect of the GM composition on disease phenotype of isogenic mouse models. For example, gastrointestinal diseases such as Inflammatory Bowel Disease (IBD), colorectal cancer, and obesity have been associated with alterations in taxa within *Clostridiaceae*, *Ruminococcaceae*, *Lachnospiraceae* and/or *Rikenellaceae*^35–39^ making the use of mice differentially colonized with taxa within these families desirable for targeted complex GM studies. In neurodevelopment models, such as models of autism where decreased relative abundance of taxa within *Prevotellaceae* are shown to be associated with disease severity^40^, CD1_GMJAX_ surrogate dams harboring a higher mean relative abundance may be desirable to investigate the contribution of taxa within this family on disease severity. In addition to evaluation of composition, the use of these standardized CD1 colonies offers the ability to examine the effect of GM diversity on disease phenotype. Several recent studies have suggested that alterations in GM complexity is associated with disease severity in a variety of diseases including IBD, obesity, and type 2 diabetes^41–45^. As the CD1 colonies have statistical differences in GM diversity, comparisons of GM diversity effects on model phenotype can be achieved using these colonies as GM donors. We previously used surrogate dams from these colonies to transfer isogenic IL-10^−/−^ embryos, in a process we call complex microbiota targeted rederivation (CMTR), to demonstrate differential effects of complex GM on disease phenotype in the IL-10^−/−^ mouse model of inflammatory bowel disease (IBD)^22^. Our results underscore the potential utility of these colonies as a means to study complex GM profiles.

The National Institutes of Health (NIH) recently raised concerns over the reproducibility and variability of rodent studies within biomedical research^46,47^. Studies have shown that alterations in the GM can result in alteration of model phenotype^9,11,48^. This finding coupled with recent studies indicating rodent producers have different GM profiles^20,49^ suggest that these factors may significantly contribute to observed within model variability. In addition to use as a tool to address specific GM targeted research questions where CMTR is warranted, these CD1 colonies should also be considered as a means to address concerns over model reproducibility. As these colonies have stable, well characterized GM profiles and good reproduction indices they should be considered for use as GM donors for other types of fecal transfer (Table 1). For example, CD1 dams are well known for their docile nature and strong maternal care instincts making them an attractive option for GM transfer by cross-fostering, whereby pups from a mouse strain with the desired genetics are removed from the dam immediately following birth and are transferred to a CD1 dam with the desired GM profile. Due to the stability and well characterized nature of the four GM profiles, these colonies can also be considered as standardized donor GM pools which can be used for additional methods of GM transfer such as methods to colonize axenic mice or the use of fecal microbiota transfer (FMT) using pre-transfer antibiotic treatment. Fecal pellets, from one or more of the CD1 colonies with the desired GM profile, could be collected and a fecal slurry could then be gavaged to axenic or antibiotic-treated mice. Alternatively, due to the docile nature of CD1 mice, GM transfer by co-housing could be implemented. With this method, young mice from one or more of the CD1 colonies could be co-housed with similarly aged, same sex, mice of the genetic model of interest.

**Table 1:** Uses of CD1 colonies with standardized, complex GM profiles.

Uses of Standardized Complex Microbiota Colonies		
	Advantage(s)	Disadvantage(s)
CMTR Surrogates	• Natural vaginal transfer of GM • Complete transfer of GM • First generation pups available for study of early life events	• Expertise for embryo transfer • High cost of rederivation/embryo transfer
GM donors for axenic mice	• Stable complex GM that represent those in contemporary colonies	• High cost of gnotobiotic studies
GM donors via fecal transplant (post antibiotic)	• Moderate cost	• Success limited to transfer of high richness GM to low richness GM (opposite transfers ineffective) • Incomplete transfer
Cross fostering surrogates	• Ease of use • Low cost • Early (w/in 24 hr) transfer of GM	• Requires timed mating • Non-vaginal GM transfer • Incomplete transfer resulting in hybridized GM • Need for second generation pups for early life events
GM donors via co-housing	• Ease of use • Low cost	• Incomplete transfer resulting in hybridized GM • Need for second generation pups for early life events

Collectively, the data in this study provide proof of concept that surgical embryo transfer to establish outbred mice with differing vendor-specific gut microbiota is possible. We have demonstrated that these GM profiles are stable for at least nine generations, underscoring the stability of the GM in apparently healthy mice. As the GM composition of these mice is a naturally occurring complex GM, they provide a unique alternative strategy to study the effect of complex GM in isogenic mouse models and can provide a method to help ensure model reproducibility.

### Ethics approval and consent to participate

The current study was conducted in accordance with the guidelines set forth by the Guide for the Use and Care of Laboratory Animals and the Public Health Service Policy on Humane Care and Use of Laboratory Animals. All studies and protocols were approved by the University of Missouri, University of California Davis, and University of North Carolina Institutional Animal Care and Use Committees.

### Availability of data and material

The datasets generated and analyzed during the current study are available at the NCBI SRA database as project number PRJNA474117.

## References

[1] Sekirov I, Russell SL, Antunes LC, Finlay BB (2010). Gut microbiota in health and disease. Physiol Rev.

[2] Pflughoeft KJ, Versalovic J (2012). Human microbiome in health and disease. Annu Rev Pathol.

[3] Cenit MC, Matzaraki V, Tigchelaar EF, Zhernakova A (2014). Rapidly expanding knowledge on the role of the gut microbiome in health and disease. Biochim Biophys Acta.

[4] Shreiner AB, Kao JY, Young VB (2015). The gut microbiome in health and in disease. Curr Opin Gastroenterol.

[5] Cryan JF, Dinan TG (2012). Mind-altering microorganisms: the impact of the gut microbiota on brain and behaviour. Nat Rev Neurosci.

[6] Ericsson AC, Franklin CL (2015). Manipulating the Gut Microbiota: Methods and Challenges. ILAR J.

[7] Franklin CL, Ericsson AC (2017). Microbiota and reproducibility of rodent models. Lab Anim (NY).

[8] Laukens D, Brinkman BM, Raes J, De Vos M, Vandenabeele P (2016). Heterogeneity of the gut microbiome in mice: guidelines for optimizing experimental design. FEMS Microbiol Rev.

[9] Atarashi K (2011). Induction of colonic regulatory T cells by indigenous Clostridium species. Science.

[10] Gaboriau-Routhiau V (2009). The key role of segmented filamentous bacteria in the coordinated maturation of gut helper T cell responses. Immunity.

[11] Ivanov II (2009). Induction of intestinal Th17 cells by segmented filamentous bacteria. Cell.

[12] Backhed F (2004). The gut microbiota as an environmental factor that regulates fat storage. Proc Natl Acad Sci USA.

[13] Lesher S, Walburg HE, Sacher GA (1964). Generation Cycle in the Duodenal Crypt Cells of Germ-Free and Conventional Mice. Nature.

[14] Thompson GR, Trexler PC (1971). Gastrointestinal structure and function in germ-free or gnotobiotic animals. Gut.

[15] Sudo N (2004). Postnatal microbial colonization programs the hypothalamic-pituitary-adrenal system for stress response in mice. J Physiol.

[16] Kibe R (2005). Movement and fixation of intestinal microbiota after administration of human feces to germfree mice. Appl Environ Microbiol.

[17] Turnbaugh PJ (2009). The effect of diet on the human gut microbiome: a metagenomic analysis in humanized gnotobiotic mice. Sci Transl Med.

[18] Bel S (2014). Reprogrammed and transmissible intestinal microbiota confer diminished susceptibility to induced colitis in TMF−/− mice. Proc Natl Acad Sci USA.

[19] Ericsson AC, Personett AR, Turner G, Dorfmeyer RA, Franklin CL (2017). Variable Colonization after Reciprocal Fecal Microbiota Transfer between Mice with Low and High Richness Microbiota. Front Microbiol.

[20] Ericsson AC (2015). Effects of vendor and genetic background on the composition of the fecal microbiota of inbred mice. PLoS One.

[21] Ericsson AC (2015). Differential susceptibility to colorectal cancer due to naturally occurring gut microbiota. Oncotarget.

[22] Hart ML, Ericsson AC, Franklin CL (2017). Differeing Complex Microbiota Alter Disease Severity of the IL-10−/− Mouse Model of INflammatory Bowel Disease. Front Microbiol.

[23] Montonye DR (2018). Acclimation and Institutionalization of the Mouse Microbiota Following Transportation. Front Microbiol.

[24] Magoc T, Salzberg SL (2011). FLASH: fast length adjustment of short reads to improve genome assemblies. Bioinformatics.

[25] Martin DD (2011). The use of bone age in clinical practice - part 2. Horm Res Paediatr.

[26] Edgar RC (2010). Search and clustering orders of magnitude faster than BLAST. Bioinformatics.

[27] Kuczynski, J. *et al*. Using QIIME to analyze 16S rRNA gene sequences from microbial communities. *Curr Protoc Bioinformatics* Chapter 10, Unit10 17, 10.1002/0471250953.bi1007s36 (2011). 10.1002/0471250953.bi1007s36 PMC3249058 22161565

[28] Edgar RC (2013). UPARSE: highly accurate OTU sequences from microbial amplicon reads. Nat Methods.

[29] Altschul SF (1997). Gapped BLAST and PSI-BLAST: a new generation of protein database search programs. Nucleic Acids Res.

[30] Pruesse E (2007). SILVA: a comprehensive online resource for quality checked and aligned ribosomal RNA sequence data compatible with ARB. Nucleic Acids Res.

[31] Hammer O, Harper D, Ryan P (2001). PAST: Paleontological statistics software package for educaiton and data analysis. Palaeontologica Electronica.

[32] Bejamini YaH, Controlling Y (1995). the False Discovery Rate: A Practical and Powerful Approach to Multiple Testing. Journal of the Royal Statistical Society, Series B (Methodological).

[33] Xia J, Sinelnikov IV, Han B, Wishart DS (2015). MetaboAnalyst 3.0–making metabolomics more meaningful. Nucleic Acids Res.

[34] Xia J, Wishart DS (2011). Web-based inference of biological patterns, functions and pathways from metabolomic data using MetaboAnalyst. Nat Protoc.

[35] Frank DN (2007). Molecular-phylogenetic characterization of microbial community imbalances in human inflammatory bowel diseases. Proc Natl Acad Sci USA.

[36] Joossens M (2011). Dysbiosis of the faecal microbiota in patients with Crohn’s disease and their unaffected relatives. Gut.

[37] Sartor RB, Mazmanian SK (2012). Intestinal Microbies inInflammatory Bowel Diseases. Am J Gastroenterol Suppl.

[38] Walters WA, Xu Z, Knight R (2014). Meta-analyses of human gut microbes associated with obesity and IBD. FEBS Lett.

[39] Peters BA (2016). The gut microbiota in conventional and serrated precursors of colorectal cancer. Microbiome.

[40] Kang DW (2013). Reduced incidence of Prevotella and other fermenters in intestinal microflora of autistic children. PLoS One.

[41] Manichanh C (2006). Reduced diversity of faecal microbiota in Crohn’s disease revealed by a metagenomic approach. Gut.

[42] Ott SJ (2004). Reduction in diversity of the colonic mucosa associated bacterial microflora in patients with active inflammatory bowel disease. Gut.

[43] Comito D, Cascio A, Romano C (2014). Microbiota biodiversity in inflammatory bowel disease. Ital J Pediatr.

[44] Turnbaugh PJ (2009). A core gut microbiome in obese and lean twins. Nature.

[45] Larsen N (2010). Gut microbiota in human adults with type 2 diabetes differs from non-diabetic adults. PLoS One.

[46] Perrin S (2014). Preclinical research: Make mouse studies work. Nature.

[47] Collins FS, Tabak LA (2014). Policy: NIH plans to enhance reproducibility. Nature.

[48] Yang I (2013). Intestinal microbiota composition of interleukin-10 deficient C57BL/6J mice and susceptibility to Helicobacter hepaticus-induced colitis. PLoS One.

[49] Hildebrand F (2013). Inflammation-associated enterotypes, host genotype, cage and inter-individual effects drive gut microbiota variation in common laboratory mice. Genome Biol.

